# Comparison of the Multiple Reaction Monitoring and Enhanced Product Ion Scan Modes for Confirmation of Stilbenes in Bovine Urine Samples Using LC–MS/MS QTRAP^®^ System

**DOI:** 10.1007/s10337-016-3121-1

**Published:** 2016-07-05

**Authors:** Iwona Matraszek-Zuchowska, Barbara Wozniak, Andrzej Posyniak

**Affiliations:** Department of Pharmacology and Toxicology, National Veterinary Research Institute, 24-100 Pulawy, Poland

**Keywords:** Stilbenes, Confirmatory method, LC–MS/MS, Multiple reaction monitoring, Enhanced product ion

## Abstract

In accordance with Commission Decision 2002/657/EC, confirmatory methods for the detection of prohibited substances should comply with specific requirements, including the criteria for confirmation. Two strategies: multiple reaction monitoring (MRM) and enhanced product ion (EPI) scanning functions were compared for confirming the anabolic compounds from synthetic stilbenes group in bovine urine samples. In the research, twenty samples fortified at the Recommended Concentration (RC) of 1 µg L^−1^ with diethylstilbestrol, dienestrol and hexestrol were analyzed by liquid chromatography-tandem mass spectrometry on a QTRAP 5500 instrument. The analytical procedure, validated in accordance with the Commission Decision 2002/657/EC, used in the official control of hormones in Poland was applied. The validation parameters were in agreement with 2002/657/EC performance criteria. The effectiveness of MRM and EPI scanning modes for confirmation purposes was evaluated based on the percentage of the results confirmed. In all urine samples recorded in the MRM mode, the confirmation criteria (retention time, relative intensities between transitions) have been fulfilled. The presence of stilbenes in all urine samples using EPI scan mode was confirmed too as evidenced by a good matching of stilbenes spectra in the samples to the reference spectra with critical match factor above 0.7. The results of the research show that EPI scanning function provides the same effectiveness for confirmation of banned compounds as the mostly used MRM scan mode and can be an additional tool to confirm the doubtful case results in the analysis of hormones residues, even at such low concentration levels.

## Introduction

In the European Union, the use of hormonal growth-promoting active drugs is prohibited for fattening purposes under Council Directive 96/22/EC in order to protect consumers against residues with possible toxic effects on public health [1–3].

For effective control of illegal use of hormones and the determination of residues in samples from the area of food safety, highly specific and sensitive analytical methods are required [4]. In addition, confirmatory methods for the detection of banned, hormonally active substances in biological samples of animal origin must be approved and meet special requirements including the criteria for identification as defined in Commission Decision 2002/657/EC, the norm obligatory in all Member States of the European Union [5, 6]. Confirmatory methods should also provide complementary information on the chemical structure of the analytes and fragmentation mechanisms and pathways. Consequently, chromatographic methods with spectrometric detection are recommended to be used for confirmation.

A number of chromatographic methods, including gas chromatography (GC) and liquid chromatography (LC) in combination with different types of mass spectrometry (MS) have been demonstrated to determine hormones residues at low concentration levels in samples of animal origin [7–12]. In recent years, LC tandem mass spectrometry (MS/MS) with triple quadrupole (QqQ) mass analyzers operating in MRM scan mode was the dominant and powerful technique due to its high sensitivity and selectivity, used to quantify the targeted analytes such as drugs, steroids and pharmaceuticals at trace amounts in a variety of matrix [13–16]. From a technical point of view, in MRM mode, the two mass filtration steps are employed on a triple quadrupole mass spectrometer. In the first stage, specific precursor ion of interest is pre-selected in the first quadrupole (Q1) and the fragmentation is induced by collision excitation with a neutral gas in a pressurized collision cell (the second quadrupole Q2). In the second stage, fragments (product ions) generated in Q2 are analyzed in the third quadrupole (Q3). The advantage of MRM based methods in principle provides both complete structural specificity of the analyte and the relative or absolute concentration measurement.

In the case of the LC–MS/MS confirmatory methods, it is required to obtain at least four identification points; the presence of one precursor and two daughter ions provides 4IPs, and the presence of two precursor ions, each with one daughter ensures 5IPs. In addition the relative intensities of the detected ions, expressed as a percentage of the intensity of the most intense transition, should correspond to the standard or spiked sample, in reference to the maximum permissible tolerance. The observation of two MRM transitions, indicating the chromatographic peak of the analyte at the expected retention time and the resulting area ratio (ion ratio) is considered solid verification criteria. Furthermore, the relative retention time of the analyte in the tested sample should be consistent with the relative retention time of the analyte in the spiked sample (or standard) with a tolerance of 2.5 %.

Recent articles in the literature, report that, despite such high requirements for confirmation, the likelihood of finding false identification, for example because of the matrix effect-well known problem can be considerably higher [17, 18]. Therefore, the search for and use additional methods and alternatives to confirm the presence of banned substances is particularly important, both to protect consumers from unwanted residues as well as manufacturers because of penalties. The equipment such as a new devices, detectors and sensors entering the market, offers innovative advanced features and appears to be a promising solution, not only in relation to such research.

The development and introduction of a new generation of LC–MS/MS systems in the form of a hybrid triple quadrupole linear ion trap spectrometers (QTRAP^®^ System) by AB SCIEX in 2002, allows thanks to this technology to combine MRM scan mode with the ion trap scanning functions. MRM scanning type is the most commonly used, target method in a conventional triple quadrupole system. Whereas the capability of an ion trap enabling fast and high scanning sensitivity by utilizing methods such as enhanced product ion scan and various other approaches for recording of the useful mass spectra of each detected signal [19, 20].

In QTRAP^®^ system in comparison to QqQ, the third quadrupole can be operated in dual function both as a quadrupole and Linear Ion Trap (LIT). This allows simultaneously detection of a compound by MRM transitions and identification on the basis of automatically acquired MS/MS ion spectra recorded at a fixed area threshold setting. For that purpose, information-dependent acquisition (IDA), a powerful extension of software is generally used. It maximizes the information content generated in a single run. The difference for LIT in technical point as compared to QqQ refers to the fact that the precursor ions and/or product ions depending on the scan mode exit the collision cell (Q2) and enter the third quadrupole Q3 operating in LIT mode. Then, ions are trapped in Q3 and after a few milliseconds fill time of accumulation, they are scanned before filtration.

Thanks to the application of ion trap scanning modes in relation to the common scanning mode of a triple quadrupole MS, the identification can be achieved with much higher sensitivity. A significant impact on it has the opportunity to register characteristic MS/MS spectra of fragment ions in a lower cycle time and their interpretation with the use of the mass spectral library searching.

Due to the fact that hormones residues are determined at very low concentration levels (of the order 1 μg L^−1^), the analysis of these compounds requires in addition to the proper sample preparation and advanced instrumental techniques that significantly improve the performance of identification and confirmation of compounds. The new generation of LC–MS/MS equipment with the possibility of combining some types of scans can be useful to enable the determination and identification of such low ppb levels of hormones in biological samples.

Up to now, the number of publications using these approaches to identify the residues of the banned anabolic compounds in the samples of animal origin remains limited. The applications of EPI scanning function are usually associated with multi-component screening analytical methods most often related to the determination of natural and plant origin substances, pesticides and different veterinary drugs also [21–26]. From the perspective of the extensive range of tested compounds, the identification by comparing the spectra with the library is fast and easy. Until now, reports on the comparison of MRM and EPI measuring techniques for confirmation of banned hormonal compounds according to the current criteria have not been published. The aim of the study was to evaluate the applicability of the EPI scanning function for confirmation of synthetic hormones from the group of stilbenes: diethylstilbestrol, dienestrol, hexestrol in the urine and compare it with the currently used conventional MRM scan mode.

## Materials and Methods

### Reagents and Chemicals

Standards of stilbenes: diethylstilbestrol (DES), dienestrol (DIE) and hexestrol (HEX) were all obtained from Sigma-Aldrich (Steinheim, Germany). The standards of labeled stilbenes: diethylstilbestrol-d6 (DES-d6), dienestrol-d2 (DIE-d2) and hexestrol-d4 (HEX-d4) were purchased from Institute of Food Safety-RIKILT (Wageningen, The Netherlands). Standard ampoules were stored at room temperature or at 2–8 °C according to the certificates. Primary standard stock solutions at a concentration of 1 mg mL^−1^ or 10 µg mL^−1^ were prepared in methanol and stored below −18 °C. Working standard solutions (at a concentration of 1 or 0.1 µg mL^−1^) were obtained by further tenfold dilutions of the stock solutions with methanol and were kept at 2–8 °C for no longer than 6 months.

Diethyl ether, concentrated acetic acid (99.5 %), hydrochloric acid (0.1 M), anhydrous sodium sulfate (99.5 %), sodium hydrogen carbonate (99.5 %), and sodium acetate anhydrous (99.0 %) were of analytical grade and were obtained from POCH (Gliwice, Poland). Sodium carbonate (99.8 %) was provided from Sigma-Aldrich (Steinheim, Germany). The n-hexane (99.5 %), methanol (99.8 %), acetone (99.4 %), all residue grade quality and methanol (99.8 %) HPLC–MS grade were obtained from Mall Baker (Deventer, The Netherlands). Purified water was achieved by using Milli-Q apparatus (Millipore, Bedford, MA, USA). β-Glucuronidase (23 U mL^−1^)/aryl sulfatase (68 U mL^−1^) Helix Pomatia and Tris(hydroxymethyl)-aminomethane buffer substance (analytical grade) were purchased from Merck (Darmstadt, Germany). Solid phase extraction (SPE) cartridges (Bakerbond^®^ C_18_ 500 mg/3 mL and NH_2_ 500 mg/3 mL) were supplied by Mall Baker (Deventer, The Netherlands).

Acetate buffer (0.05 M), pH 4.8 was prepared by mixing 40 mL of 0.05 M solution of acetic acid (1.42 mL in 500 mL of water) with 60 mL of 0.05 M solution of sodium acetate (4.1 g in 1000 mL of water) and adjusting the pH to 4.8. Acetate buffer (0.04 M), pH 5.2 was prepared by mixing 25 mL of 0.04 M solution of acetic acid (1.2 mL in 500 mL of water) with 75 mL of 0.04 M solution of sodium acetate (3.28 g in 1000 mL of water) and determining the value of pH to 5.2. Tris buffer (20 mM), pH 8.5 was prepared by dissolving Tris solid substance (4.8 g) in water (500 mL), and then mixing 50 mL of this solution with 9 mL of hydrochloric acid (0.1 M), next diluting with water to 200 mL, and adjusting pH to 8.5. 10 % sodium hydrogen carbonate and 10 % sodium carbonate solutions in water were prepared by dissolving solid substances (100 g) in water (900 mL). Carbonate buffer was prepared by mixing 10 % sodium hydrogen carbonate solution (100 mL) with 10 % sodium carbonate solution (500 mL) and determining the pH to 10.25.

### LC–MS/MS (MRM and EPI) Measurement

HPLC analysis was performed using an autosampler, a column oven and a binary pump system (1200 series, Agilent Technologies, Waldbronn, Germany). For the separation of stilbenes a Poroshell 120 EC-C18 analytical column (150 mm × 2.1 mm, 2.7 µm) (Agilent Technologies, Waldbronn, Germany) with octadecyl guard cartridge (4 mm × 2 mm) (Phenomenex, Torrance, CA, USA) has been applied. The mobile phase was a mixture of a methanol/water (70:30, v/v) pumped in isocratic mode at a total flow rate set to 140 μL min^−1^. The column was maintained at a constant temperature of 45 °C. The injection volume was 25 μL.

For MS/MS analysis, QTRAP 5500 mass spectrometer (Applied Biosystems/MDS SCIEX, Toronto, Canada) based on the conventional triple quadrupole ion path with the properties of an ion-trap for the third quadrupole, controlled by Analyst Software (version 1.6) for data collection and processing was used. The following conditions of detection were applied: an electrospray ionization (ESI) Turbo Spray source operating in negative mode at 250 °C with the appropriate settings: curtain gas (nitrogen) 20 psi, nebulizer gas (air) 20 psi, auxiliary gas (air) 25 psi, collision gas (nitrogen) at medium position, ionization voltage −4500 V, MRM dwell time 40 ms, pause between mass range 5 ms and entrance potential (EP) −10 V. For the tested compounds the following transitions under optimal instrumental conditions of collision energy (CE), declustering potential (DP) and collision cell exit potential (CXP) were obtained: for DES: 267.0 > 237.2 (CE = −38 eV, DP = −140 V, CXP = −10 V), 267.0 > 222.2 (CE = −46 eV, DP = −140 V, CXP = −11 V), 267.0 > 209.2 (CE = −50 eV, DP = −140 V, CXP = −10 V); for DES-d6: 273.3 > 237.2 (CE = −40 eV, DP = −140 V, CXP = −12 V); for DIE: 265.1 > 236.2 (CE = −30 eV, DP = −150 V, CXP = −9 V), 265.1 > 93.0 (CE = −34 eV, DP = −150 V, CXP = −12 V); for DIE-d2: 266.8 > 93.1 (CE = −34 eV, DP = −150 V, CXP = −10 V); for HEX: 269.0 > 133.0 (CE = −23 eV, DP = −100 V, CXP = −10 V), 269.0 > 119.0 (CE = −59 eV, DP = −100 V, CXP = −12 V) and for HEX-d4: 273.3 > 121.1 (CE = −56 eV, DP = −100 V, CXP = −13 V).

MRM transitions selected for stilbenes were used to construct an EPI survey scans in IDA experiment with mass spectral library search. The total scan time (including pauses) was 0.2918 s for all MRM transitions. Each transition was performed with a dwell time of 40 ms and pause time of 1.5 ms; MS/MS EPI spectra were registered at following three values of collision energy (CE): −32, −43 and −45 eV and Collision Energy Spread (CES) of 15 V. The IDA dependent scan intensity threshold was set to 10,000 cps for DES, 5000 cps for HEX and 30,000 cps for DIE, respectively. Dynamic exclusion of 60 s and the mass tolerance of 250 mDa were applied. Fragments formed in the product ion spectra were detected in the range between 50 and 330 amu with dynamic fill time and a scan rate of 10,000 Da s^−1^, and the resolution of Q1 device set to unit.

### Sample Preparation

Five mL of urine, centrifuged and filtered using membrane filters (25 mm, 0.45 μm) for clarification of aqueous solutions (Millex^®^-HA, Millipore, Bedford, Ireland) were adjusted to pH 5.2 by adding of a few droplets of glacial acetic acid, if needed. To the sample 5 mL of acetate buffer was added and 5 µL of deuterated internal standards (DES-d6, DIE-d2 and HEX-d4) at a concentration of 1 µg mL^−1^ to obtain a final concentration of 1 μg L^−1^. Next, an enzymatic hydrolysis (37 °C ± 2 °C, overnight) with glucuronidase AS–HP (50 μL) was performed. The digested sample was cooled to the room temperature and was extracted twice with 20 and 10 mL of diethyl ether. The collected organic layers were washed with 20 mL of carbonate buffer and 20 mL of distilled water, dried on anhydrous sodium sulfate and evaporated under the gentle stream of nitrogen at 60 °C (±2 °C). The residue was dissolved in 3 mL of acetate buffer (0.05 M, pH 4.8) and applied onto C_18_ SPE column previously conditioned with 3 mL of methanol and 3 mL of TRIS buffer/methanol mixture (80:20, v/v). The column was washed with 3 mL of TRIS buffer/methanol mixture (80:20, v/v) next with 3 mL of methanol/water mixture (45:55, v/v) and stored under vacuum. The stilbenes were eluted with 3 mL of acetone and the eluate was directly loaded on SPE NH_2_ column previously conditioned with 5 mL of methanol/water mixture (80:20, v/v). The eluate was collected in glass tube and evaporated to dryness under the gentle stream of nitrogen at 60 °C (±2 °C). Finally, the residue was reconstituted in 100 μL of mobile phase consisting of methanol–water mixture (70:30, v/v) and aliquots of the solution (25 μL) were analyzed using QTRAP 5500 LC–MS/MS system operated in MRM and EPI scan modes.

### Method Parameters

The LC–MS/MS quantitative and confirmatory method applied in this study is used for official residue control of stilbenes in Poland. The method has been validated in accordance with the requirements specified in Commission Decision 2002/657/EC [5] and ISO/11843 2000 approach [27].

The following validation parameters such as instrumental linearity, specificity, repeatability, reproducibility, recovery, decision limit (CCα), detection capability (CCβ), the uncertainty and ruggedness were estimated.

For the factorial effect analysis the recommended software “ResVal” (v 2.0) (CRL Laboratory, The Netherlands) was used [28]. The instrumental linearity was evaluated on the basis of 4 standard calibration curves prepared for each of the trials in the mobile phase, drawing in eight points, containing a fixed amount of internal standards (1 μg L^−1^ each), with analytes concentrations corresponding to 0, 0.1, 0.2, 0.5, 1.0, 2.0, 4.0 and 6.0 μg L^−1^ in a sample. In the validation process, one hundred and eleven of cattle urine samples have been globally included. Sequentially, three series of samples were performed (experiment 1–3). Each of them containing a blank reference sample, 6 samples spiked at concentration levels of 0.5, 1.0 and 1.5 μg L^−1^, one sample spiked at concentration of 2.0 μg L^−1^ as well as one sample spiked at concentration level of 5.0 μg L^−1^. On the basis of these three experiments matrix matched calibration curves were constructed from which the CCα and CCβ values were calculated by ResVal software according to the approach described in the ISO/11843 2000 [27]. The calculation of CCα and CCβ was based on the following mathematical equations:

1 $$ {\text{CC}}\alpha  = ((y_{a} + 2.33 \cdot {\text{STD}}_{a} ) - y_{a} ))/b $$

2 $$ {\text{CC}}\beta  = ((y_{a} + 2.33 \cdot {\text{STD}}_{a} + 1.64 \cdot {\text{STD}}_{a} ) - y_{a} ))/b $$

in which the *y*_*a*_ defines the intercept of calibration curve, the STD_*a*_ specifies the standard deviation of the *y*_*a*_, and *b* mean slope of calibration curve. Twenty urine samples spiked at the estimated CCα were prepared, to check the reliability of these values [29].

The influence of the matrix on the signal response was investigated based on the ratio of the slopes of standard and matrix-matched calibration curves [21].

Apparent recovery was assessed in relation to the deuterium labeled internal standards. The specificity study was evaluated from the analysis of 10 different blank urine samples taken from bovine and porcine and simultaneously the same 10 samples of urine fortified at 1.0 μg L^−1^.

According to this validation software, the expanded uncertainty (*U*) was calculated as the sum of variances of reproducibility on levels 0.5–1.5 μg L^−1^ and variance of the matrix effects using a coverage factor (*k*) of 2.

For all samples tested during validation process, the criteria for confirmation required for LC–MS/MS QqQ MRM method by Commission Decision 2002/657/EC were verified. The presence of stilbenes was confirmed in more than 95.0 % of the samples examined.

The method has been successfully verified during participation in two FAPAS Proficiency Tests: round of Synthetic Hormones and Thyreostats in Bovine Urine and Synthetic Hormones in Bovine Urine which were carried out in 2012. Our *z* score results obtained for dienestrol and diethylstilbestrol were −0.4 and −0.7, respectively.

Calibration parameters and performance of the method for the determination of diethylstilbestrol, dienestrol and hexestrol in the urine at the concentration level of 1 µg L^−1^ are presented in Table 1.

**Table 1:** Equations calibration curves and results of the method validation for the DES, DIE, HEX in urine samples at a concentration 1 µg L^−1^

Compound	DES	DIE	HEX
Calibration curve of standard			
Linear range	0.1–6 µg L^−1^		
Slope ± *s*_*b*_	2.30 ± 0.25	1.30 ± 0.26	1.46 ± 0.22
*y* intercept ± *s*_*a*_	−0.32 ± 0.19	−0.07 ± 0.20	−0.19 ± 0.17
Linear correlation coefficient (*r*)	0.9990	0.9994	0.9985
Coefficient of determination (*r* ^2^)	0.9980	0.9988	0.9970
Standard error	0.248	0.109	0.187
Matrix-matched calibration curve			
Linear range	0.07–5 µg L^−1^		
Slope ± s_b_	2.09 ± 0.32	1.3 ± 0.4	1.29 ± 0.17
*y* intercept ± s_a_	−0.04 ± 0.27	0.11 ± 0.11	0.04 ± 0.16
Linear correlation coefficient (*r*)	0.9999	0.9999	0.9998
Coefficient of determination (*r* ^2^)	0.9998	0.9998	0.9996
Standard error	0.062	0.019	0.047
Apparent recovery (%) (*n* = 18)	103.5	110.5	107.0
Repeatability (R.S.D., %) (*n* = 18)	6.7	4.0	2.6
Within-lab reproducibility (R.S.D., %) (*n* = 18)	14.1	12.6	3.4
Decision limit (CCα, μg L^−1^)	0.07	0.07	0.07
Detection capability (CCβ, μg L^−1^)	0.12	0.12	0.11
Measurement uncertainty at 1μg L^−1^ (*U*, *k* = 2)	0.30	0.32	0.22
Matrix effect (%)	−9.1	0.0	−11.6
Samples fulfilling confirmation criteria at 1 µg L^−1^(%) (*n* = 28)	100.0	100.0	95.8

*n* number of samples tested

### The Research Material

The study was conducted on twenty different samples of bovine urine free of hormones (Reference Samples of Blank Urine BOV01-20, from EURL-RIKILT) fortified with synthetic stilbenes: diethylstilbestrol, dienestrol and hexestrol at 1 µg L^−1^ (RC) concentration level [30].

## Results and Discussion

For the compounds being the subject of research, the confirmatory criteria required for the MRM and EPI scan modes were checked. In the MRM the number of IP obtained for all analytes was consistent with the requirements of the current legislation. For diethylstilbestrol one precursor ion and three daughters corresponding ions were obtained whereby 5.5 IPs has been achieved, that exceeded the minimum required number of 4 IPs. For the remaining two tested compounds: dienestrol and hexestrol, for each one precursor ion with two daughters corresponding ions were obtained which yielded 4 IPs.

With respect to the guidelines of the 2002/657/EC permitted tolerances for relative ions intensities for DES, DIE and HEX, on the basis of standards solutions were established. The MRM transitions used for identification of stilbenes and transitions ratios both in standards solutions and in the twenty samples of urine as well as the results of confirmation of these compounds are presented in the Table 2. As is apparent from the cited table, the relative intensities of the ions for DES, DIE and HEX in twenty spiked urine samples were contained within the specified ranges designated for standards.

**Table 2:** The confirmation results of DES, DIE and HEX in twenty bovine urine samples spiked at 1 µg L^−1^, registered in the MRM mode

Compound	MRM transition (*m*/*z*)	Average relative ion intensity ± SD for standards	Average relative ion intensity ± SD for urine spiked	Samples fulfilling confirmation criteria (%)
DES	**267.0** **>** **237.2** 267.0 > 222.2 267.0 > 209.2	0.866 ± 0.007 0.266 ± 0.031	0.866 ± 0.018 0.284 ± 0.013	100.0 100.0
DES-d6	**273.3** **>** **237.2**	–	–	–
DIE	265.1 > 236.2 **265.1** **>** **93.0**	0.17 ± 0.05	0.161 ± 0.002	100.0
DIE-d2	**266.8** **>** **93.1**	–	–	–
HEX	269.0 > 133.0 **269.0** **>** **119.0**	0.487 ± 0.008	0.491 ± 0.008	100.0
HEX-d4	**273.3** **>** **121.1**	–	–	–

^a^The most intense transitions in bold used for quantification

Additionally the compatibility of the relative retention time of stilbenes in the samples and in the twenty registered standards solutions within the specified tolerance range has been confirmed.

The results obtained in the studies indicate that the presence of DES, DIE and HEX in all twenty spiked samples of urine taking into account all the provisions relating to the retention time and relative intensities of transitions has been confirmed.

The second way to confirm the presence of the stilbenes in the urine sample was the application of the EPI scan mode offered by Triple quadrupole linear ion trap QTRAP^®^ system.

Although, according to the Commission Decision 2002/657/EC, the identification of compounds can be carried out using computer-aided library searching and the abundance of the fragment ions should be greater than 10 % of the intensity of the most intense ion of the spectra but the criteria of matching spectra have not been specified.

Because no commercial library of spectra of stilbenes was available in-house library reference spectra of compounds was constructed manually.

**Fig. 1:**
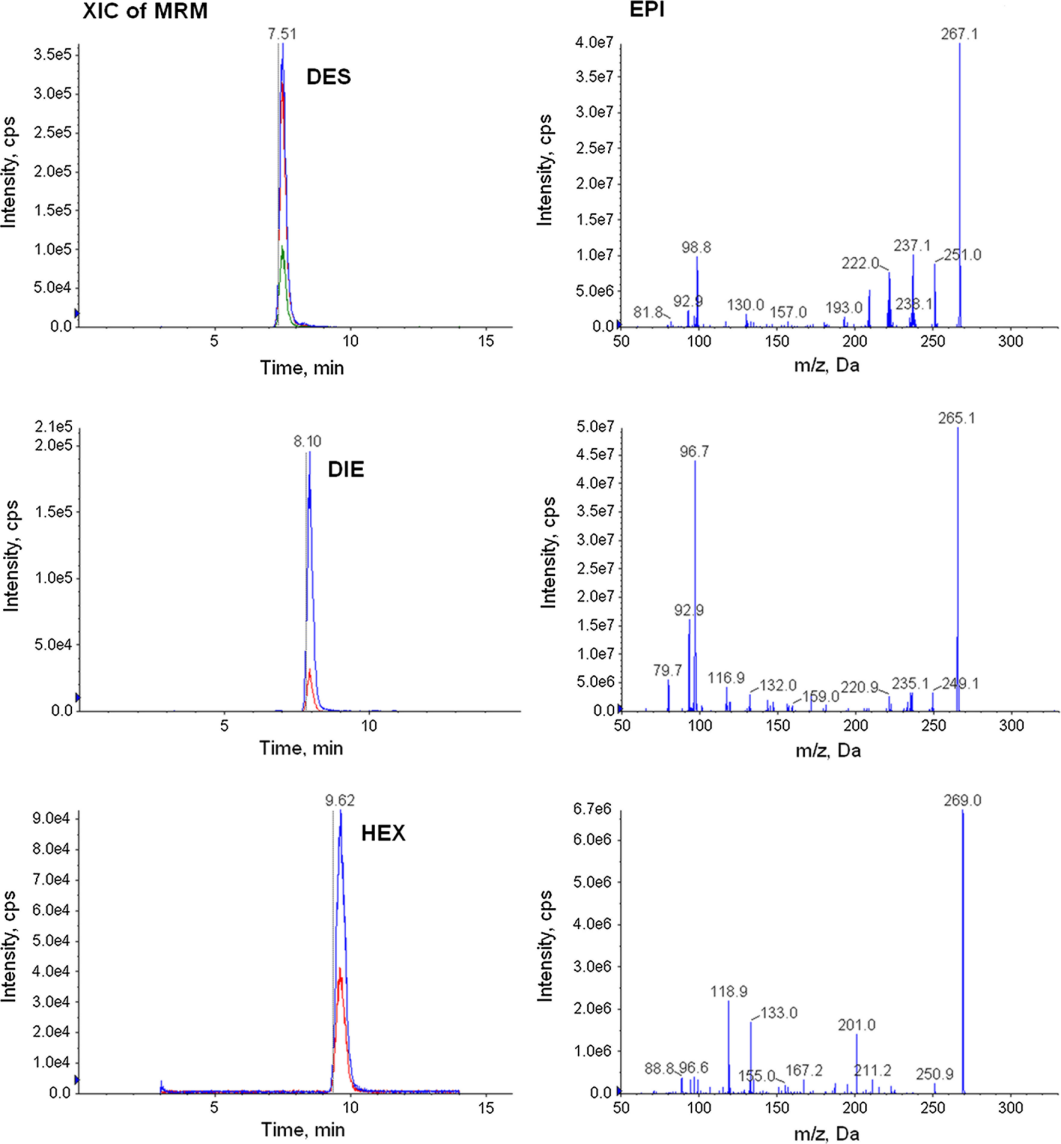
The XIC of MRM chromatograms and EPI spectra of DES (CE −43; 10,000 cps), DIE (CE −40; 30,000 cps) and HEX (CE −32; 5000 cps) in standard solution corresponding to 1 μg L^−1^ in urine sample

The library created was based on the EPI spectra registered at the three indicated values of CE for each stilbenes dosing individually in a standard solution with a concentration of analytes corresponding to 0.1–5 μg L^−1^ in the sample. During the registration of stilbenes spectra, IDA threshold and CE conditions particularly affecting the fragmentation have been tested and determined as optimal. XIC of MRM chromatograms and EPI spectra of DES, DIE and HEX in the standard solution corresponding to 1 μg L^−1^ in urine sample, under optimal conditions of CE and IDA threshold are presented in Fig. 1.

In the first stage EPI spectra of stilbenes in twenty standards solutions in the concentration range corresponding to 0.1–1 µg L^−1^ in the urine sample were searched against the existing in-house mass spectral library and reproducibility of the spectra was monitored. Similarly, EPI spectra of stilbenes registered in the twenty spiked urine samples of cattle were searched against the existing in-house mass spectral library. Then the degree of similarity of the spectra was evaluated. The matching spectra were assessed based on the criteria of the following three fit indexes: fit value (Fit), reverse fit value (RevFit) and purity fit (Purity) factor given by the software.

Fit provides information on the similarity of signals in the reference library spectrum with those in the registered spectrum whereas RevFit reflects the similarity of signals in the registered spectrum with those in the reference spectrum. Finally, Purity, which characterizes the spectral similarity, is a combination of both Fit and RevFit values and measures unmatched peaks between the registered spectra and librarian.

The results of library search of spectra recorded in EPI scan mode are presented in Table 3. Reproducibility of stilbenes spectra presented as coefficients of variations for individual fit indexes fall within the range of 2.3–8.0 % in standards solutions while in the spiked urine samples within the range of 1.7–9.2 %, indicating optimal performance. The determined differences between Fit, RevFit and Purity factors in standards solutions and matrix were lower than 5 % as shown in the table discussed. It can be concluded that the three examined fit indexes may be used as a criterion for the identification of stilbenes in urine samples.

**Table 3:** Statistics of library search results of Fit, RevFit and Purity for stilbenes obtained in twenty standard solutions with a concentration of DES, DIE and HEX corresponding to 0.1–1 μg L^−1^ in the urine sample and in urine samples spiked at 1 μg L^−1^

Analyte	Fit index	Standard		Matrix		Mean_standard_ − Mean_matrix_ (%)	CV_standard_ − CV_matrix_
		Mean	CV (%)	Mean	CV (%)		
DES	Fit	0.89	8.0	0.94	4.0	−5.68	3.9
	RevFit	0.94	5.0	0.87	7.7	6.63	−2.7
	Purity	0.85	7.4	0.84	8.5	0.93	−1.0
DIE	Fit	0.97	3.5	0.98	1.7	−0.77	1.8
	RevFit	0.98	2.3	0.96	4.0	2.30	−1.7
	Purity	0.96	4.4	0.95	4.3	0.78	0.0
HEX	Fit	0.86	7.3	0.91	3.5	−6.33	3.8
	RevFit	0.91	5.3	0.86	9.2	4.88	−3.9
	Purity	0.83	7.4	0.82	7.5	0.70	−0.1

In principle, according to the QTRAP software permissible values of Fit and RevFit indexes describing the similarity between unknown EPI spectrum and the reference one in the library should be greater than 0.5.

However, with respect to the statistics, the convergence factor, one of the key measures of quality and indicating the degree of matching, should be greater than 0.9; perfect match gives a score of 1. So library hit with Fit, RevFit and Purity values above 0.9 has excellent identification. From a statistical point of view, the convergence factors in the range of 0.8–0.9 and in the range of 0.7–0.8 indicate a good and satisfactory matching, respectively. Therefore, in our study, the value of 0.7 has been proposed as the cut-off values for fit matching indexes to ensure accurate identification. Other authors in their studies of compounds different than hormones and at much higher concentration levels accepted the same value of fit coefficient for matching spectra [19, 20, 31].

For stilbenes being the subject of the research in all standards solutions and all spiked samples individual values of Fit, RevFit and Purity indexes were not less than 0.7 values. Whereas all determined mean of the fit indexes values for stilbenes in standards and spiked samples were greater than 0.8 (Table 3) and indicate both a good matching and proper identification of analytes.

By applying the principles of statistics on the degree of identity of spectra, the population of twenty samples tested in which the presence of the stilbenes has been confirmed has been properly grouped. As presented in Table 4, taking into account both Fit and RevFit indexes in all urine samples, the presence of DES and HEX was confirmed with a very good and good matching of spectra for at least 80 % of samples and about 20 % with satisfactory under to the accepted criteria. The best matching (100 %) was obtained for DIE, spectrum of which is less specific than the spectra of the other compounds. Considering separately Purity index, representing empirical indicator, combining the impact of Fit and RevFit factors, the percentage of results confirmed was very similar [21]. The presence of stilbenes with a very good and good matching of spectra was confirmed also for at least 80 % of samples for DES, for 80 % of samples for HEX and for 100 % of samples for DIE. Summing up the results obtained it can be concluded that in accordance with the assumptions of statistics stilbenes have been confirmed in all of the analyzed samples.

**Table 4:** The confirmation results of DES, DIE and HEX in twenty bovine urine samples spiked at 1 µg L^−1^, registered in the EPI mode

Compound	Samples fulfilling confirmation criteria			
	Fit ≥ 0.9 RevFit ≥ 0.9 Purity ≥ 0.9	0.9 > Fit ≥ 0.8 0.9 > RevFit ≥ 0.8 0.9 > Purity ≥ 0.8	0.8 > Fit ≥ 0.7 0.8 > RevFit ≥ 0.7 0.8 > Purity ≥ 0.7	(%)
DES	45.0	35.0	20.0	100.0
	25.0	55.0	20.0	
DIE	95.0	5.0	–	100.0
	90.0	10.0	–	
HEX	35.0	50.0	15.0	100.0
	–	80.0	20.0	

Fit; RevFit ≥ 0.9, Purity ≥ 0.9—very good matching of spectra to reference. 0.9 > Fit; RevFit ≥ 0.8, 0.9 > Purity ≥ 0.8—good matching of spectra to reference. 0.8 > Fit; RevFit ≥ 0.7, 0.8 > Purity ≥ 0.7—satisfactory matching of spectra to reference

The results presented in Tables 2 and 4 proves that both MRM and EPI scan modes are suitable for the identification of hormones on such low concentration levels.

When it is suspected that the sample contains an illegal growth promoter, avoiding false-positive results is a priority over. For this reason it is important to have reliable confirmatory methods in the analysis of banned compounds.

Therefore, as shown, the efficacy of EPI scan mode is exceptionally important tool that provide additional functionality for the identification purposes in complicated analyzes and it can be used to confirm prohibited compounds at low levels. In the opinion of H. F. De Brabander, despite the existence of objective criteria to identify banned compounds presented in the document 2002/657/EC, it could be risky treated GC or LC–MS/MS methods as error-free. The author emphasizes, that only two independent research using different techniques, which gives the same qualitative and quantitative results, guarantee the reliability of the results [32]. Thus, the search for additional ways of identification of anabolic compounds seems to be valid.

## Conclusion

Selection of appropriate methods for the analysis of residues in many cases not only depends on the type of problem, but also on the ultimate goal. In order to ensure food safety it is important to avoid false-negative and false-positive findings which have serious legal and financial consequences. To minimize the potential risk of incorrect interpretation of results, the methods giving greater confidence in hormones identification should be used.

The results of research showed that both MRM and EPI detection techniques have the same effectiveness in confirmation of stilbenes in urine samples even at such low level of 1 µg L^−1^.

On this basis, it can be stated that the use of EPI measurement may be an effective and useful strategy for identifying banned compounds as well as widely used MRM scan mode.

Therefore enhanced product ion scanning function can in particular be used to correct the questionable multiple reaction monitoring results in analysis of hormones residues. The application of EPI scan mode to confirm the presence of banned stilbenes in urine has been described for the first time.
